# Novel norovirus recombinants and of GII.4 sub-lineages associated with outbreaks between 2006 and 2010 in Belgium

**DOI:** 10.1186/1743-422X-8-310

**Published:** 2011-06-18

**Authors:** Elisabeth Mathijs, Sarah Denayer, Leonor Palmeira, Nadine Botteldoorn, Alexandra Scipioni, Alain Vanderplasschen, Etienne Thiry, Katelijne Dierick

**Affiliations:** 1 Department of Infectious and Parasitic Diseases, Veterinary Virology and Animal Viral Diseases, Faculty of Veterinary Medicine, University of Liege, Liege, Belgium; 2 Department of Infectious and Parasitic Diseases, Immunology and Vaccinology, Faculty of Veterinary Medicine, University of Liege, Liege, Belgium; 3 Communicable and Infectious Diseases, Food borne Pathogens, Scientific Institute of Public Health, Brussels, Belgium

## Abstract

**Background:**

Noroviruses (NoVs) are an important cause of acute gastroenteritis in humans worldwide. To gain insight into the epidemiologic patterns of NoV outbreaks and to determine the genetic variation of NoVs strains circulating in Belgium, stool samples originating from patients infected with NoVs in foodborne outbreak investigations were analysed between December 2006 and December 2010.

**Results:**

NoVs were found responsible of 11.8% of all suspected foodborne outbreaks reported in the last 4 years and the number of NoV outbreaks reported increased along the years representing more than 30% of all foodborne outbreaks in 2010. Genogroup II outbreaks largely predominated and represented more than 90% of all outbreaks. Phylogenetic analyses were performed with 63 NoV-positive samples for the partial polymerase (N = 45) and/or capsid gene (N = 35) sequences. For 12 samples, sequences covering the ORF1-ORF2 junction were obtained. A variety of genotypes was found among genogroups I and II; GII.4 was predominant followed in order of importance by GII.2, GII.7, GII.13, GI.4 and GI.7. In the study period, GII.4 NoVs variants 2006a, 2006b, 2007, 2008 and 2010 were identified. Moreover, phylogenetic analyses identified different recombinant NoV strains that were further characterised as intergenotype (GII.e/GII.4 2007, GII.e/GII.3 and GII.g/GII.1) and intersub-genotype (GII.4 2006b/GII.4 2007 and GII.4 2010/GII.4 2010b) recombinants.

**Conclusions:**

NoVs circulating in the last 4 years in Belgium showed remarkable genetic diversity either by small-scale mutations or genetic recombination. In this period, GII.4 2006b was successfully displaced by the GII.4 2010 subtype, and previously reported epidemic GII.b recombinants seemed to have been superseded by GII.e recombinants in 2009 and GII.g recombinants in 2010. This study showed that the emergence of novel GII.4 variants together with novel GII recombinants could lead to an explosion in NoV outbreaks, likewise to what was observed in 2008 and 2010. Among recombinants detected in this study, two hitherto unreported strains GII.e/GII.3 and GII.g/GII.1 were characterised. Surveillance will remain important to monitor contemporaneously circulating strains in order to adapt preventive and curative strategies.

## Background

Noroviruses (NoVs) are on the rise as a causative agent of gastroenteritis in humans of all ages and are responsible for approximately 90% of epidemic non-bacterial outbreaks of gastroenteritis in the world. Transmission can occur through direct contact with shedding persons, contaminated food, sewage-contaminated water, contaminated aerosols and environmental contamination [1]. Food-borne transmissions have been estimated to account for 14% of infections due to NoV [2]. The major issue with NoV infections is their frequent occurrence in large outbreaks in community settings such as hospitals and nursing homes [3,4].

The genus *Norovirus *belongs to the family *Caliciviridae *along with 4 other genera (*Vesivirus, Lagovirus, Sapovirus and Nebovirus*). NoVs are non-enveloped viruses with a single-stranded, positive-sense, polyadenylated RNA genome of about 7500 nucleotides (nt) in length [5]. Three overlapping ORFs encode the non-structural (ORF1) and structural (ORF2 and ORF3) viral proteins. The ORF1-encoded polyprotein is cleaved further by the viral proteinase into six mature products including the genome-linked virus protein (VPg), the proteinase and the highly conserved RNA-dependent RNA-polymerase [6]. Due to its high conservation, the polymerase has been a widely used target for molecular detection assays [7]. ORF 2 encodes the major capsid protein (VP1) that contains an N-terminal arm, a shell or S-domain and a protrusion or P-domain. The P-domain is divided into 2 sub-domains called P1 and P2, the latter corresponding to the most variable region of the capsid. ORF3 encodes for a small minor structural protein of the virion [8].

NoVs are genetically highly diverse and are divided into five genogroups (GI-V) which are further subdivided into at least 29 genotypes based on genetic differences in the capsid gene [9-11]. GI and GII NoVs are responsible for most human infections. The absence of a regular cell-culture system or small-animal model for human NoVs led to the development of molecular tools to study the epidemiology of NoVs. GII.4 has been by far the most detected genotype being responsible for 60 to 70% of the outbreaks globally reported to the Foodborne Viruses in Europe (FBVE) network between 1990 and 2008 [12]. During this same period, 4 large pandemics (1995-1996, 2002, 2004-2005 and 2006) have been identified corresponding each time to the emergence of one or two new variants of the GII.4 lineage and the displacement of the previously predominant circulating ones [13,14]. Frequently reported as second most prevalent NoVs after GII.4 strains, are GII.b recombinants [15]. Since the first detection in France in August 2000, they have been involved in outbreaks across the globe thereby demonstrating their spreading capacity among human populations [16,17]. Along with small-scale mutations, recombination events seem to contribute to the genetic variability between NoVs. This phenomenon was identified by incongruent clustering of different regions of the genome in phylogenetic analyses for NoVs from all 5 genogroups [18-20]. Based upon predictive models, the majority of NoV recombinants have breakpoints located either within or close to the ORF1-ORF2 junction. Consequently, this region has been suggested to constitute a recombination hotspot in NoVs [21].

To gain insight into the epidemiologic patterns of NoV outbreaks and to determine the genetic variation of NoVs strains circulating in Belgium, we analyzed stool samples originating from patients infected with NoVs in foodborne outbreak investigations conducted by the Belgian Scientific Institute of Public Health (IPH). These studies involved predominantly large outbreaks occurring in community settings between December 2006 and December 2010.

## Methods

### Definitions

At European level a food-borne outbreak (FBO) is defined as an incidence, observed under given circumstances, of two or more human cases of the same disease and/or infection; or a situation in which the observed number of human cases exceeds the expected number and where the cases are linked, or are probably linked, to the same food source (Directive 2003/99/EC, Article 2(d)). A human NoV infection is described as a case with the typical gastroenteritis symptoms as started between 12-24 h after infection, nausea, vomiting, diarrhoea and in some cases also slight fever along with the laboratory confirmation of the presence of NoVs in clinical samples.

### Sample collection and stool specimens

Since 2005, the IPH harbors the National Reference Laboratory for foodborne outbreaks (NRL FBO) and it is responsible for collecting, centralizing the information, reporting and follow up of FBOs in Belgium. In case of an outbreak, stool samples were sent to the IPH by the Health Inspectors or clinical laboratories at hospitals. Food samples were sent to the NRL FBO by the local inspectors of the Federal Agency for Safety of the Food Chain (FASFC). Samples were stored refrigerated (2-8°C) during transport and analyses started the day of arrival at the NRL FBO. In total, 164 NoV-positive stool specimens from cases of acute gastro-enteritis were collected during the 4-year period from December 2006 through December 2010.

### Laboratory investigation: norovirus detection

Stool suspensions (10%, [weight/volume]) were prepared in phosphate buffered saline (PBS). One hundred microliters supernatant was collected after centrifugation for 5 minutes at 13000 rpm (Eppendorf 5415D, Rotselaar, Belgium). RNA was extracted using the RNeasy Mini Kit (Qiagen, Leusden, The Netherlands) following the manufacturer's instructions. NoV was extracted from foodstuff according to the protocol as previously described with slight modifications [22]. Briefly, 10 g of food was homogenized with 8 ml TRIzol^® ^reagent (Invitrogen, Merelbeke, Belgium) and shaken for 20 min at room temperature to allow contact. After centrifugation for 10 minutes (13000 rpm) at 4°C (Eppendorf 5804R, Rotselaar, Belgium) the aqueous phase is transferred into a new tube for further concentration/purification. Two hundred microliters of chloroform is added for each ml of TRIzol^® ^reagent used, and mixed for 15s followed by 2-3 min settling before centrifugation (13000 rpm, 15 min, 4°C). One hundred microliters of the upper (aqueous) phase was used for RNA extraction using the RNeasy Mini Kit (Qiagen, Leusden, The Netherlands) following the manufacturer's instructions. The reverse transcription (RT) step was performed using the Transcriptor High Fidelity cDNA synthesis kit (Roche, Vilvoorde, Belgium) according to the manufacturer's instructions. Real-Time PCR (qPCR) was performed using available primers and probes for the detection of the majority of the human GI (QNIF4, CGCTGGATGCGNTTCCAT; NV1LCR, CCTTAGACGCCATCATCATTTAC; NV1LCpr, FAM-TGGACAGGAGAYCGCRATCT-TAMRA) and GII (QNIF2, ATGTTCAGRTGGATGAGRTTCTCWGA; COG2R, TCGACGCCATCTTCATTCACA; QNIFS, FAM-AGCACGTGGGAGGGCGATCG-TAMRA) NoV strains, recommanded by the CEN/TC/WG6/TAG4 research group [23,24]. Double stranded DNA (plasmid DNA GI and plasmid DNA GII) was used as positive control of the qPCR at a concentration of 2500 copies per reaction [25].

### RNA preparation, genomic amplification for genotyping

For all samples tested positive for norovirus by RT-qPCR, viral RNA was re-extracted from 140 μl sample supernatant with the QIAamp viral RNA mini kit (Qiagen, Leusden, The Netherlands) according to the manufacturer's instructions. RNA extracts were stored at -80°C before use. For genotyping, published primers were used for the amplification and sequencing of the 3' of the polymerase gene (region A in ORF1), the 5' end of the capsid gene (region C in ORF2) and the nearly full length major capsid gene [7,26,27] (Table 1 and 2). First-stranded cDNA was generated by an iScript cDNA Synthesis kit (Bio-Rad, Nazareth, Belgium). PCRs were carried out on 4 μl cDNA in 50 μl nuclease-free water containing 300 nM of both forward and reverse primers, 0.1 mM dNTPs, 2.5% DMSO, 20 mM Tris/HCl, 10 mM (NH4)2SO4, 10 mM KCl, 2 mM MgSO4, 0.1% Triton X-100 and 1 U *Taq *DNA Polymerase (New England Biolabs, Leusden, The Netherlands). The nearly complete capsid gene and ORF1-ORF2 junction were amplified using Iproof High Fidelity polymerase (Biorad, Nazareth, Belgium). Sequences covering the overlap between ORF1 and ORF2 were amplified when phylogenetic analyses indicated incongruent clustering for the partial sequences of the polymerase and the capsid genes for the same samples. After amplification, the amplicons were visualized by electrophoresis and purified by using either a standard ethanol precipitation protocol or the QIAquick PCR purification kit (Qiagen, Leusden, The Netherlands) according to instructions given by the manufacturer. Direct sequencing of PCR products was carried out by GATC Biotech sequencing facilities (Konstanz, Germany) in both directions using an ABI 3730xl DNA Analyzer (Applied Biosystems, Lennik, Belgium).

**Table 1:** Characteristics of genotyped norovirus-positive clinical samples

Year	Sample Name	Outbreak code	Outbreak localisation	Date of first symptoms	Sequenced region	Genotyping result
2006	IPH55	HAI0016	Hainaut	22/12/2006	A	GII.4 2006b
	IPH57				A+C	GII.4 2006b
	IPH59				A	GII.4 2006b

2007	IPH472	-	Namur	10/04/2007	A+C	GII.4 2006a
	IPH473				A+C	GII.4 2006a
	IPH474				A+C	GII.4 2006a
	IPH475				A+C	GII.4 2006a
	IPH477				A+C	GII.4 2006a

2008	IPH2159	08VG06	Limburg	30/09/2008	A	GII.2
	IPH2161				Junction	GII.2
	IPH2162				Junction	GII.2
	
	IPH2204	08VG01	Flemish Brabant	1/10/2008	Junction	GII.4 2008
	IPH2206				Junction	GII.4 2008
	
	IPH2252	VBR008	Flemish Brabant	30/09/2008	C	GI.4
	IPH2255				Junction	GI.4
	
	IPH2700	08VG07	East Flanders	14/11/2008	A+C	GII.4 2006b/GII.4 2007
	
	IPH2839	OVL009	East Flanders	26/11/2008	Junction A	GII.e/GII.4 2007 GII.4 2006b/--
	
	IPH3007	ANT005	Antwerp	2/12/2008	A+C	GII.7
	
	IPH3059	08VG08	Limburg	12/12/2008	A	GII.4 2006b
	IPH3060				A+C	GII.4 2006b
	IPH3064				A	GII.4 2006b
	IPH3069				A+C	GII.e/GII.4 2007

2009	IPH143	09VG2	Limburg	6/02/2009	Junction	GII.e/GII.4 2007
	
	IPH608	09VG3	Limburg	22/03/2009	Junction	GII.4 2006b
	
	IPH2171	09VG4	Limburg	05/05/2009	A+C	GII.e/GII.3
	IPH2172				Junction	GII.e/GII.3
	IPH2174				A+C	GII.e/GII.3
	IPH2175				Junction	GII.e/GII.3
	IPH2176				A+C	GII.e/GII.3
	
	IPH3075	ANT011	Antwerp	8/12/2009	A	GII.4 2010/--

2010	IPH159	10VG1	Limburg	27/01/2010	A	GII.4 2010/--
	IPH161				A+C	GII.4 2010
	
	IPH163	LIM001	Limburg	27/01/2010	Junction	GII.g/GII.1
	IPH164				A+C	GII.g/GII.1
	
	IPH165	10VG2	Limburg	1/02/2010	A+C	GII.4 2010/GII.4 2010b
	IPH167				A	GI.7/--
	IPH251				A+C	GII.4 2010/GII.4 2010b
	
	IPH294	10VG3	Brussels	2/02/2010	A+C	GII.4 2010/GII.4 2010b
	IPH296				A+C	GII.4 2010/GII.4 2010b
	IPH297				A+C	GII.4 2010/GII.4 2010b
	IPH298				A+C	GII.4 2010/GII.4 2010b
	IPH301				A+C	GII.4 2010/GII.4 2010b
	
	IPH310	10VG9	Antwerp	17/02/2010	C	--/GII.4 2010b
	IPH312				A+C	GII.4 2010/GII.4 2010b
	IPH314				A+C	GII.4 2010/GII.4 2010b
	
	IPH317	10VG4	Antwerp	17/02/2010	A+C	GII.4 2010/GII.4 2010b
	
	IPH505	10VG5	Antwerp	04/03/2010	A	GII.g/--
	
	IPH506	10CF1	Namur	04/03/2010	A+C	GII.4 2010/GII.4 2010b
	IPH671				A+C	GII.4 2010/GII.4 2010b
	
	IPH748	LIM003	Limburg	05/04/2010	C	--/GII.13
	
	IPH966	LIM005	Limburg	08/05/2010	A+C	GII.g/GII.1
	IPH967				A+C	GII.g/GII.1
	IPH968				A	GII.g/--
	
	IPH1010	ANT003	Antwerp	8/05/2010	A+C	GII.4 2010
	
	IPH1093	OVL005	East Flanders	19/05/2010	A	GII.7/--
	
	IPH1143	ANT004	Antwerp	18/05/2010	A+C	GII.2
	
	IPH1919	VBR006	Flemish Brabant	17/07/2010	A+C	GII.g/GII.1
	IPH1936				A+C	GII.g/GII.1
	
	IPH1920	LIM007	Limburg	17/07/2010	C	--/GII.1
	
	IPH2020	LIM008	Limburg	27/07/2010	A+C	GII.g/GII.1
	IPH2021				A+C	GII.g/GII.1
	IPH2022				A+C	GII.g/GII.1
	IPH2045				Junction	GII.g/GII.1

A: partial polymerase gene; C: partial capsid gene; Junction: ORF1-ORF2 overlap

**Table 2:** Oligonucleotide primers used for genotyping

Region	Name	Sequence (5' → 3')^a^	Sense	Position^b^	Reference
A	290d	GATTACTCCASSTGGGAYTCMAC	+	4568-4590	[50]
A	289d	TGACGATTTCATCATCMCCRTA	-	4865-4886	
A	JV12Y	ATACCACTATGATGCAGAYTA	+	4552-4572	[7]
A	JV13I	TCATCATCACCATAGAAIGAG	-	4858-4878	
C	G1SKF	CTGCCCGAATTYGTAAATGA	+	5342-5361	[26]
C	G1SKR	CCAACCCARCCATTRTACA	-	5652-5671	
C	G2SKF	CNTGGGAGGGCGATCGCAA	+	5046-5064^c^	
C	G2SKR	CCRCCNGCATRHCCRTTRTACAT	-	5367-5389^c^	
Capsid	FW1	GCGATCGCAATCTGGCTCCCAG	+	5055-5076^d^	[27]
Capsid	RT5	AGGTGYACATTATGACCAGTTC	-	6795-6819^d^	

^a ^International Union of Biochemistry ambiguity codes: I, inosine; N, (A/C/T/G); H, (A/C/T); Y, (C/T); M, (A/C); R, (A/G); S, (C/G).

^b ^Position in the norovirus genomic sequence GI.1 (M87661)

^c ^Position in the norovirus genomic sequence GII.1 (U07611)

^d ^Position in the norovirus genomic sequence GII.4 (AY032605)

### Molecular typing, phylogenetic analysis and recombination study

All sequences were typed with the genotyping tool for NoVs, National Institute of Public Health and the Environment, The Netherlands at http://www.rivm.nl. Database searches for related sequences were conducted using BLAST. Reference strains for phylogenetic analysis were selected from the Norovirus genotyping tool and are given in the Additional file 1 Table S1. All sequences were aligned at the protein level using the MUSCLE algorithm [28] to produce more robust alignments and were back-translated into nucleic acid sequences for subsequent analyses. Phylogenetic trees were inferred on the previously aligned sequences under a maximum-likelihood framework using PhyML [29] under a GTR evolutionary model [30] with optimized invariable sites and a discrete gamma model with 4-classes of optimized rates to model the variability of substitution rates across sites [31]. Branch support was inferred using the Shimodaira-Hasegawa-like non-parametric procedure [32]. To accommodate for weak phylogenetic signal, a thorough exploration of the tree space was made through topological rearrangements using a combination of Nearest Neighbor Interchange and Subtree Pruning and Regrafting topology search methods as implemented in PhyML [33,34]. Finally, for visualization purposes, the trees were then arbitrarily rooted.

### Nucleotide sequence accession numbers

Accession numbers for sequences obtained for partial polymerase gene, partial capsid gene and/or partial polymerase and capsid gene with the overlapping junction of the ORF1-ORF2 junction in this study are: [GenBank: EU794891-EU794894; EU794879-EU794883; JF697202-JF697293].

## Results

### Characterisation of NoV outbreaks in Belgium, 2006-2010

**Figure 1:**
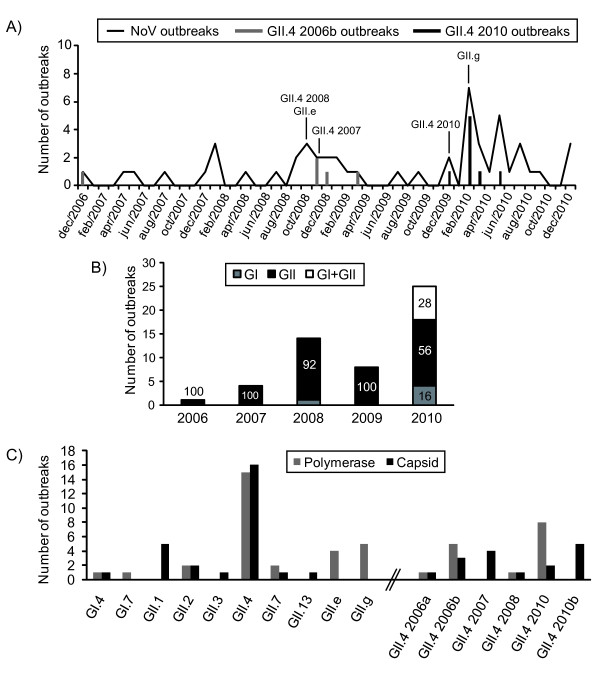
**Characteristics of noroviruses detected in acute gastroenteritis outbreaks reported in Belgium**. A) Monthly distribution of norovirus (NoV)-associated outbreaks in Belgium from December 2007 to December 2010. Months corresponding to the primer detection of novel NoV genotypes (GII.e and GII.g) or sub-lineages (GII.4 variants 2007, 2008 and 2010) are indicated by an arrow; Diversity of NoV genogroups (B), genotypes and GII.4 sub-lineages (C) detected in gastro-enteritis outbreaks in Belgium during 2006 and 2010. Percentages of genogroup prevalence are indicated in the vertical bars. Typing results were obtained by phylogenetic clustering with reference strains (http://www.noronet.nl) either for the partial polymerase gene sequence (grey) or the partial capsid gene sequence (black).

From December 2006 to December 2010, a total of 458 gastroenteritis outbreaks, suspected to be food-borne, were reported to the Belgian IPH (Additional file 2 Table S2). During this period, NoVs were involved in 54 (11.8%) of the reported outbreaks and affected 34.5% of all patients reported ill. Classification of NoVs into genogroups GI and GII was possible for all samples tested positive by RT-qPCR but more accurate genotyping (genotype and sub-genotype) was only possible for over half of the NoV outbreaks (Additional file 2 Table S2). Indeed, phylogenetic analyses were performed with 63 NoV-positive samples for the partial polymerase (N = 45) and/or capsid gene (N = 35) sequences. For 12 samples, sequences covering the ORF1-ORF2 junction were obtained. Despite the fact that NoVs were detected in some food samples, no sequences could be obtained as amplification by conventional RT-PCR was unsuccessful. With the exception of 2009, the number of reported NoV outbreaks increased throughout the years; particularly in 2010 where NoV outbreaks represented more than 30% of the reported outbreaks. When the monthly distribution of NoV outbreaks over the 4-year study period was plotted, two peaks in NoV activity could be seen; one in the autumn-winter 2008/2009 and another from December 2009 to September 2010 (Figure 1A). In both cases, the increase in NoV activity coincided with the detection of novel NoV strains. From 2006 until 2010, GII NoVs outbreaks largely predominated and represented 90.4% of the NoVs either in single infections or in co-infection with GI NoVs (Figure 1B). GI single infections and GI-GII co-infections represented respectively 16 and 28% of the outbreaks reported in 2010. Phylogenetic analysis allowed GII NoVs clustering into 7 different genotypes: GII.4 strains (15 outbreaks, 50%), recombinant GII.g/GII.1 (5 outbreaks, 16.7%), recombinant GII.e/GII.4 (3 outbreaks, 10%), GII.2 (2 outbreaks, 6.6%), GII.7 (2 outbreaks, 6.6%), GII.13 (1 outbreak, 3.3%) and recombinant GII.e/GII.3 (1 outbreak, 3.3%) (Figure 1C). Two GI NoV outbreaks were successfully genotyped and clustered into GI.4 and GI.7. Figure 1C also shows the variety of GII.4 variants identified since 2006: 2006a, 2006b, 2007, 2008 and 2010; of all the GII.4 variants the majority clustered with the new GII.4 2010 variant. The only outbreak reported in 2006 was linked with GII.4 2006b whereas GII.4 2006a was identified in the only typed outbreak of 2007. The 2006b variants were detected until March 2009 in co-circulation with GII.4 variants 2007 and 2008; subsequently they were displaced by the 2010 variant first described in December 2009 (Figure 1A).

### Detection of novel GII.4 variants

**Figure 2:**
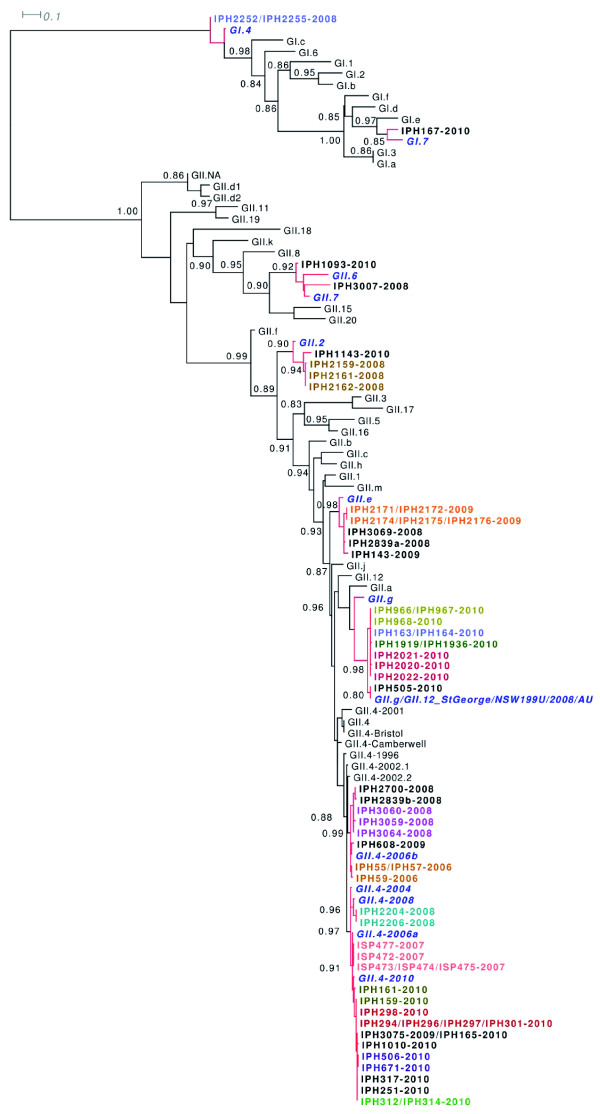
**Phylogenetic analyses of the partial polymerase region of the detected norovirus genomes**. Phylogenetic trees were inferred under a maximum-likelihood framework from the nucleic acid sequences aligned at the protein level (GTR model with aLRT node support, see Material and Methods for details). The aLRT node supports were only indicated when superior to 0.8 and relevant to the genotype identification. Reference NoV strains identified as highly related to the norovirus (NoV) samples were highlighted in bold, italic blue font. NoV samples originating from the same outbreak and co-localized in the phylogenetic tree were also highlighted in color. Identical NoV sequences were represented on the same node (in color if originating from the same outbreak, in black if originating from different outbreaks). Details on the genotyping of each NoV sample can be found in Table 1.

**Figure 3:**
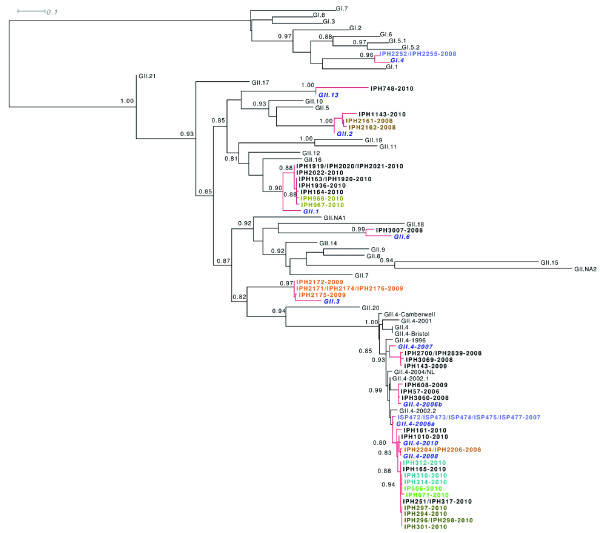
**Phylogenetic analyses of the partial capsid region of the detected norovirus genomes**. Phylogenetic trees were inferred under a maximum-likelihood framework from the nucleic acid sequences aligned at the protein level (GTR model with aLRT node support, see Material and Methods for details). The aLRT node supports were only indicated when superior to 0.8 and relevant to the genotype identification. Reference NoV strains identified as highly related to the norovirus (NoV) samples were highlighted in bold, italic blue font. NoV samples originating from the same outbreak and co-localized in the phylogenetic tree were also highlighted in color. Identical NoV sequences were represented on the same node (in color if originating from the same outbreak, in black if originating from different outbreaks). Details on the genotyping of each NoV sample can be found in Table 1.

Phylogenetic clustering with GII NoV reference strain sequences from the Noronet European genotyping tool (Additional file 1 Table S1) allowed the characterisation of NoV circulating during the period of this study. Phylogenetic trees were drawn for both the partial polymerase and capsid gene sequences (Figure 2 and 3) and genotyping results for the amplified region of each sample are shown in table 1. GII.4 NoV sequences were shown to group within 4 and 6 clusters for the polymerase and capsid genes respectively. Besides the pandemic variants GII.4 2006a and GII.4 2006b, GII.4 sequences clustered with newly reported GII.4 sequences; 2007 (for the capsid gene only), 2008 and 2010. One single outbreak in 2008 was caused by GII.4 2008 variant whereas GII.4 2007 capsid gene sequences were amplified from samples originating from 4 outbreaks in late 2008 and early 2009. In two of these outbreaks, the 2007 variant was detected with the 2006b variant either in the same sample (IPH2839) or in two samples from the same outbreak (IPH3060 and IPH3069). In a sample from a third outbreak, a GII.4 2006b polymerase was found in combination with a GII.4 2007 capsid gene sequence (IPH2700). A GI.7 NoV was found co-circulating with GII.4 2010 in samples originating from an identical outbreak. Since December 2009, GII.4 2010 was the only GII.4 variant detected. Phylogenetic analyses showed that all GII.4 2010 sequences amplified from the polymerase gene grouped into one single cluster whereas the corresponding partial capsid gene sequences formed two different genetic clusters. Of these capsids, only two (IPH161 and IPH1010) belonged to the cluster formed by the GII.4 2010 reference strain. Although highly related (97.2 and 98% similarity between the 253 bp long partial capsid gene sequences), the other strains grouped into a novel GII.4 capsid variant that we called GII.4 2010b.

### Identification of a novel GII recombinant

**Figure 4:**
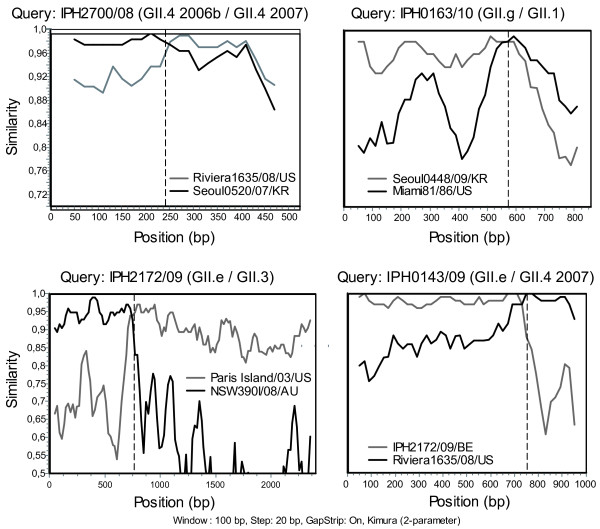
**Similarity plots of norovirus GII recombinants IPH2700/08 (A), IPH0163/10 (B), IPH2172/09 (C) and IPH0143/09 (D)**. SimPlot analyses were conducted with partial polymerase and capsid gene sequence alignments. Sequences, either a concatemer of partial polymerase and capsid sequences (A) or sequences covering the ORF1/2 overlap (B-D) were analyzed for recombination. Graphs show the similarity of the putative parental reference strains with the recombinant GII NoVs detected in our study relative to the genomic position (bp). Dashed vertical lines indicate the start of the capsid gene. Genbank accession numbers from parental NoV strains are as follows: Miami81/1986/US (GII.1): AF414416; NSW390I/2008/AU (GII.4 2007): GQ845369; Paris Island/2003/US (GII.b/GII.3): AY652979; Riviera1635/2008/US (GII.4 1996/GII.4 2007): GQ413969; Seoul0448/2009/KR (GII.g/GII.12): HM635104; Seoul0520/2007/KR (GII.4 2006b): FJ913975.

The identification of inconsistent genotype or sub-genotype clustering for the partial polymerase and capsid gene sequences for NoV strains (Figure 2 and 3) allowed the description of 5 types of recombinants involved with 14 outbreaks: GII.4 2010/GII.4 2010b (5 outbreaks), GII.g/GII.1 (4 outbreaks), GII.e/GII.4 2007 (3 outbreaks), GII.e/GII.3 (1 outbreak) and GII.4 2006b/GII.4 2007 (1 outbreak). Except from the GII.4 intergenotype recombinants, recombinants identified in this study had GII polymerase genes for which no associated capsid genes have been described, namely GII.e and GII.g which shared 93.1 - 94.2% and 95.6 - 100% nucleotide identity with reference strains OC07138/07/JP and NSW199U/08/AU, respectively. GII.e polymerases were either detected in association with GII.4 2007 or GII.3 capsids (Figure 2; Figure 3; Figure 4C and 4D) whereas all GII.g polymerases were identified with GII.1 capsids (Figure 2; Figure 3 and 4B). Simplot analyses showed potential recombination cross-over for all the recombinant types to be located at, immediately upstream or downstream the ORF1-ORF2 overlap (Figure 4).

## Discussion

During the monitoring of NoVs implicated with outbreaks in Belgium, the detection of novel GII recombinants and of GII.4 variants came along with increased outbreak reporting in 2008 and 2010. This is the first report of GII.e/GII.3 and GII.g/GII.1 recombinants implicated in outbreaks and molecular data obtained in this study for NoVs could be representative of the current epidemiological situation for Western European countries.

NoVs implicated in suspected foodborne outbreaks reported in Belgium between December 2006 and December 2010 emphasised their importance and implication in public health. Considering that, similarly to previously published data [35], the aetiology remains unknown for 20 to 50% of the reported outbreaks (data not shown), the implication of NoV in 11.8% of the outbreaks showed the importance of NoVs in foodborne outbreaks in Belgium. The presence of NoV in food was confirmed in 16 of the 35 (46%) suspected foodborne outbreaks for which food samples were provided (data not shown). Unfortunately, no sequences could be obtained from these matrices. Also, epidemiological information never involved the consumption of primary infected foodstuffs in outbreaks caused by NoV [36]. Thus, outbreaks analysed in the study period mainly consisted in NoV outbreaks where human-to-human transmissions were involved either by the consumption of food contaminated by a foodhandler or by direct contact. Hence, NoVs detected from these outbreaks reflect the panel of NoVs circulating among individuals in a contemporaneous population.

As commonly reported in the literature, GII NoVs largely predominated (90.4% of all outbreaks) and GII.4 genotype NoVs were identified in 16 of the 29 typed outbreaks (55.2%). Phylogenetic analyses indicated that GII NoV strains detected in Belgium formed different clades and even subclusters in the pylogenetics trees, especially for GII.4 NoVs (Figure 1C, 2 and 3). GII.4 subtyping identified the circulation of 5 different variants since the winter of 2006 namely 2006a, 2006b, 2007, 2008 and 2010. GII.4 2008 was identified in one single outbreak in 2008 and disappeared the same year. This GII.4 2008 variant has been associated at its primary description in Germany with a very severe case of gastroenteritis in a young boy [37]. Further data on the success of this sub-lineage in Europe and Asia are rather contradictory and report either discreet circulation [38,39] or stark implication [40,41] in epidemics for similar study periods. GII.4 2006b variants were repeatedly reported until 2009 in co-circulation with variants 2007 and 2008 before being displaced by newly emerged GII.4 2010 variants the following year. Multiple reports showed that until 2009, GII.4 2006b variants predominated upon the newly emerged GII.4 2007 and 2008 variants in other countries [38,42]. The emergence of the GII.4 2008 sub-lineage did not lead to a larger disease burden during the following winter season suggesting that some GII.4 variants do not dispose of selective advantages allowing their massive spread and longer persistence. In Belgium, GII.4 2010 constituted the only GII.4 variant circulating in 2010 and was involved in 8/18 typed outbreaks reported in the winter 2009-2010. Since its first detection in December 2009, the GII.4 2010 variant seemed to have definitively displaced the 2006b sub-lineage suggesting a perfect candidate for the generation of the observed new burden in NoV epidemics in 2010. Highly similar GII.4 2010 variants were identified in France as early as February 2009 [40], and also in Asia, Germany and Hungary since 2008 (BLAST analyses, data not shown) indicating its dissemination might have been widespread. More surveillance data from other continents will be needed to determine the implication of the GII.4 2010 sub-lineage in global epidemics. In this 4-year study period a diversity of GII.4 subvariants was observed with the subsequent emergence of three GII.4 variants. Our results clearly support that GII.4 variants evolve more rapidly than any other genotypes as suggested by the reported 5 to 36-fold higher mutation rate in GII.4 compared to non GII.4 strains [43].

NoV outbreaks were not exclusively linked with GII.4 viruses, 6 distinct genotypes were identified for both the polymerase (GII.g, GII.e, GII.2, GII.7, GI.4 and GI.7) and the capsid (GII.1, GII.2, GII.3, GII.7, GII.13 and GI.4) genes. Phylogenetic analysis of partial sequences of the polymerase and of the N-terminal conserved region of the capsid protein resulted in trees with different topologies for 5 different NoV strains, suggesting that recombination had occurred within these viruses. Four novel GII intergenotype and intersub-genotype recombinants (GII.e/GII.3, GII.g/GII.1, GII.4 2006b/GII.4 2007 and GII.4 2010/GII.4 2010b) were detected along with 1 previously published recombinant (GII.e/GII.4 2007). Since the end of 2008, GII.e and GII.g polymerase gene sequences were amplified in samples from 9 outbreaks in association with GII.3 or GII.4 2007 and GII.1 capsid gene sequences, respectively. Previously published sequences covering the ORF1-ORF2 junction showed that similar polymerase gene sequences have been described exclusively in association with GII.4 2007 capsids for GII.e [44-46] and GII.12 capsids for GII.g [41,44]. The fact that GII.e and GII.g recombinants identified in this study harboured other capsids than previously described might indicate that they emerged after undergoing a recent recombination event. As previously described for GII.b recombinants, several different capsid sequences can be associated with the GII.e and GII.g polymerase genes [17]. The association of these polymerases with distinct capsid gene sequences could provide them some selective advantages over monophylogenic strains. Indeed, GII.e and GII.g were both the second most prevalent genotype after GII.4 in 2008-2009 and 2010, respectively. These observations suggest that the emergence of the GII.e and GII.g recombinant NoVs could partly explain the increase in NoV activity observed for these periods. Data upon the NoVs implicated with the latest NoV epidemics across the world will be needed to confirm the importance of these polymerases in NoV epidemiology. The origin of these polymerases, however, still remains unclear as no parental full-length sequences yet have been detected.

An intersub-genotype GII.4 2006a/GII.4 2007 recombinant was described based upon a concatemer of partial polymerase and capsid gene sequences. Direct sequencing did not show any sign of co-infection but in the absence of a sequence covering the ORF1-ORF2 junction, this could not be ruled out. GII.4 2006b and GII.e/GII.4 2007 were shown to co-circulate in some 2008 outbreaks offering an opportunity for recombination in case of a multiple NoV infection. A recent study showed that part of the GII.4 genotypes circulating in Japan were in fact mosaics of former GII.4 sub-lineages [46]. Consequently, genetic recombination, in combination with small-scale mutations, is believed to play a role in the divergent evolution of GII.4 NoVs. A second potential intersub-lineage recombinant was identified based upon discordant tree topologies between the partial polymerase and capsid gene sequences of some GII.4 2010 variants. Indeed, in these samples all polymerase sequences clustered into one single group whereas the capsid gene sequences grouped into 2 distinct clusters. In the absence of a reference strain for the partial capsid gene sequences that did not cluster with GII.4 2010, they were referred to as GII.4 2010b. Although, sequencing of whole capsid genes would allow accurate characterisation of this novel variant. In this study, the highly conserved region C in the capsid gene was amplified for nearly all strains and only few full capsid gene sequences were successfully amplified. Amplifying long sequences from clinical samples remains challenging and in this study efforts were focused on the obtaining of sequences covering the ORF1-ORF2 overlap for the study of recombination breakpoints. SimPlot analyses confirmed all recombination points to be located at this junction which corresponds to the previously proposed hotspot for NoV recombination [21,47].

The nomenclature used in our study is based upon the clustering with NoV reference strains given for the NoV genotyping tool of the Dutch National Institute of Public health and the Environment made available by Noronet [48]. Great discrepancy is observed for NoV genotypes in the literature in particular for GII.4 sub-lineages that are commonly named after the year of detection. For instance, NoV strains related to the GII.4 2007 variants detected in this study were referred to as Cairo & Osaka 2007 in France and Australia [40,44], GII.4 2007a in Japan [46], GII.4 2008a in Canada [49] and cluster C in India [15]. Therefore, a common language still needs to be adopted in order to compare data between research teams. We have chosen reference strains provided by Noronet because one of the aims of the Noronet network is the elaboration of a well founded standardized nomenclature for NoVs.

## Conclusions

Although highly diverse, NoVs circulating in Belgium in the past 4 years were predominantly GII.4 sub-lineages and GII NoV recombinants indicating that both the accumulation of mutations and genetic recombination constitute major driven forces in NoV evolution. Furthermore, the emergent new GII.4 variants or GII recombinants might have had an impact on the magnitude of NoV epidemics suggesting that these strains dispose of some selective advantages over other circulating NoV strains.

A comprehensive study of the NoVs strains predominantly and contemporaneously involved in large and cost-effective gastro-enteritis outbreaks would help targeting the development of vaccines, therapeutic strategies and/or preventive measure on these particular strains.

## Competing interests

The authors declare that they have no competing interests.

## Authors' contributions

EM together with AS performed the molecular biology on NoV-positive faecal samples for sequencing, genotyping and bioinformatics. EM drafted the manuscript. SD and NB jointly collected clinical samples from outbreaks, gathered information about the outbreaks and detected NoV presence by performing RT-qPCR. LP performed all bioinformatic analyses (alignments, phylogeny and recombination studies). AV, ET and KD were responsible for the supervision of the study. All authors read and approved the final manuscript.

## Supplementary Material

Additional file 1 **Table 1 - Reference sequences used for phylogenetic analysis (http://www.noronet.nl)**. Click here for file

Additional file 2 **Table 2 - Food-borne gastroenteritis outbreaks reported in Belgium between 2006 and 2010**. Click here for file
