# Supplementary material for: Novel norovirus recombinants and of GII.4 sub-lineages associated with outbreaks between 2006 and 2010 in Belgium
Source: Virol J. 2011 Jun 18;8:310. doi: 10.1186/1743-422X-8-310 (PMC3135559; doi:10.1186/1743-422X-8-310)
Supplement: Additional file 1 — Table 1 - Reference sequences used for phylogenetic analysis (http://www.noronet.nl). [file 1743-422X-8-310-S1.DOC]

| Norovirus genotype | Polymerase gene |  | Capsid gene |  |
| --- | --- | --- | --- | --- |
|  | Strain name | Accession number | Strain name | Accession number |
|  |  |  |  |  |
| GI.1 | Norwalk/68/US | M87661 | Norwalk/68/US | M87661 |
| GI.2 | Southampton/91/UK | L07418 | Southampton/91/UK | L07418 |
| GI.3 | VA98115/98/US | AY038598 | Desert Shield395/90/UK | U04469 |
| GI.4 | Chiba407/87/JP | AB042808 | Chiba407/87/JP | AB042808 |
| GI.5 | Musgrove/89/UK | AJ277614 | Musgrove/89/UK | AJ277614 |
|  | - | - | SzUG1/JP | AB039774 |
| GI.6 | Hesse3/97/DE | AF093797 | Hesse3/97/DE | AF093797 |
| GI.7 | Winchester/94/UK | AJ277609 | Winchester/94/UK | AJ277609 |
| GI.8 | Boxer/01/US | AF538679 | Boxer/01/US | AF538679 |
| GI.a | Desert Shield395/90/UK | U04469 | - | - |
| GI.b | WUG1/JP | AB081723 | - | - |
| GI.c | SzUG1/JP | AB039774 | - | - |
| GI.d | Vesoul576/03/FR | EF529738 | - | - |
| GI.e | Chatellerault709/04/FR | EF529737 | - | - |
| GI.f | Otofuke/79/JP | AB187514 | - | - |
| GII.1 | Hawaii/71/US | U07611 | Hawaii/71/US | U07611 |
| GII.2 | Melksham/94/UK | X81879 | Melksham/94/UK | X81879 |
| GII.3 | Toronto/91/CA | U02030 | Toronto/91/CA | U02030 |
| GII.4 Bristol | Bristol/93/UK | X76716 | Bristol/93/UK | X76716 |
| GII.4 Camberwell | Camberwell | AF145896 | Camberwell | AF145896 |
| GII.4 1996 | Sydney348/97/AU | DQ078829 | 408/97003012/1996/FL | AF080558 |
| GII.4 2001 | Kaiso030556/03/JP | AB294779 | Kaiso030556/03/JP | AB294779 |
| GII.4 2002 | Farmington Hills/02/US | AY502023 | Farmington Hills/02/US | AY502023 |
| GII.4 2002 CN | Houston/TCH186/02/US | EU310927 | Houston/TCH186/02/US | EU310927 |
| GII.4 2004 | Inba/050590/05/JP | AB294785 | GII.4/04/NL | AY883096 |
| GII.4 2006a | Yerseke38/2006/NL | EF126963 | Yerseke38/2006/NL | EF126963 |
| GII.4 2006b | DenHaag89/2006/NL | EF126965 | DenHaag89/2006/NL | EF126965 |
| GII.4 2007 | - | - | OC07138/07/JP | AB434770 |
| GII.4 2008 | OC08086/08/JP | AB491291 | Apeldoorn317/2007/NL | AB445395 |
| GII.4 2010 | OB2009166 | noGenbank | OB2009166 | noGenbank |
| GII.5 | MOH/99/HU | AF397156 | Hillingdon/90/UK | AJ277607 |
| GII.6 | SaitamaU16/JP | AB039778 | Seacroft/90/UK | AJ277620 |
| GII.7 | SaitamaU4/JP | AB039777 | Leeds/90/UK | AJ277608 |
| GII.8 | SaitamaU25/JP | AB039780 | Amsterdam98-18/98/NL | AF195848 |
| GII.9 | - | - | VA97207/97/US | AY038599 |
| GII.10 | - | - | Erfurt/546/00/DE | AF427118 |
| GII.11 | Sw918/97/JP | AB074893 | swine43/JP | AB126320 |
| GII.12 | 5017.34/03/JP | EU187437 | Wortley/90/UK | AJ277618 |
| GII.13 | - | - | Fayetteville/98/US | AY113106 |
| GII.14 | - | - | M7/99/US | AY130761 |
| GII.15 | Hiroshima/66-1110/06/JP | AB360387 | J23/99/US | AY130762 |
| GII.16 | Neustrelitz260/00/DE | AY772730 | Tiffin/99/US | AY502010 |
| GII.17 | Briancon870/04/FR | EF529741 | CS-E1/02/US | AY502009 |
| GII.18 | OH-QW101/03/US | AY823304 | OH-QW101/03/US | AY823304 |
| GII.19 | OH-QW170/03/US | AY823306 | OH-QW170/03/US | AY823306 |
| GII.20 | Leverkusen267/2005/DE | EU424333 | Luckenwalde591/02/DE | EU33815 |
| GII.21 | - | - | IF1998/03/IQ | AY675554 |
| GII.NA1 | YURI/JP | AB083780 | YURI/JP | AB083780 |
| GII.NA2 | - | - | Chiba/040502/04/JP | AJ844470 |
| GII.a | Arg320 | AF190817 | - | - |
| GII.b | Pont de Roide 673/04/FR | AY682549 | - | - |
| GII.c | Snow Mountain virus | AY134748 | - | - |
| GII.d1 | Hokkaido/133/03/JP | AB212306 | - | - |
| GII.d2 | Hokkaido/34B/00/JP | AB231337 | - | - |
| GII.e | OC07138/07/JP | AB434770 | - | - |
| GII.f | S63/99/FR | AY682550 | - | - |
| GII.g | GoulburnValley5175/83/AU | DQ379714 | - | - |
|  | NSW199U/08/AU | GQ845370 |  |  |
| GII.h | OC97007/97/JP | AB089882 | - | - |
| GII.j | E3/97/GR | AY682552 | - | - |
| GII.k | OC96065/96/JP | AF315813 | - | - |
| GII.m | Pune/PC24/06/IN |  | - | - |
