# Supplementary material for: Novel norovirus recombinants and of GII.4 sub-lineages associated with outbreaks between 2006 and 2010 in Belgium
Source: Virol J. 2011 Jun 18;8:310. doi: 10.1186/1743-422X-8-310 (PMC3135559; doi:10.1186/1743-422X-8-310)
Supplement: Additional file 2 — Table 2 - Food-borne gastroenteritis outbreaks reported in Belgium between 2006 and 2010. [file 1743-422X-8-310-S2.DOC]

|  |  |  | |  | |  | | Year |  | |  | | |
| --- | --- | --- | --- | --- | --- | --- | --- | --- | --- | --- | --- | --- | --- |
|  | | | 2006 | | 2007 | | 2008 | | | 2009 | | 2010 | Total |
| GE outbreaks | | | 116 | | 75 | | 104 | | | 96 | | 67 | 458 |
| Ill individuals in GE outbreaks | | | 1032 | | 846 | | 841 | | | 857 | | 1346 | 4922 |
| Norovirus outbreaks (%)^a^ | | | 4/116 (3.4) | | 10/75 (13.3) | | 11/104 (10.6) | | | 8/96 (8.3) | | 21/67 (31.3) | 54/458 (11.8) |
| Ill individuals in NoV outbreaks^b^ (%) | | | 154/1032 (14.9) | | 392/846 (46.3) | | 439/841 (52.2) | | | 95/857 (11) | | 616/1346 (45.8) | 1696/4922 (34.5) |

^a^ Percentages are referred to the total of reported gastroenteritis (GE) outbreaks

^b^ Percentages are referred to the number of ill individuals in reported GE outbreaks

^c^ Percentages are referred to the total of reported norovirus (NoV) outbreaks
